# Hospital Affairs in Ireland

**Published:** 1919-09-27

**Authors:** 


### HOSPITAL AFFAIRS IN IRELAND.

### Radical Reforms Called For.

It's an ill wind that blows nobody good, and the refusal of the Government to increase the grant to the House of Industry Hospitals may, immediately or ultimately, have the effect of reforming some of the methods of Dublin medical practitioners.

These hospitals, it will be remembered, were established in 1772, and originally combined the functions of poorhouse, prison, penitentiary, and hospital. They are virtually, if not actually, Government property, and receive an annual grant of £7,600, but no public subscriptions, nor any share of the Hospital Sunday Fund. Owing to the decline in the purchasing power of the sovereign, they have fallen on evil days, and within recent weeks threatened to close. The Secretary, however, has now announced that they will carry on. The public are invited to subscribe, but the real departure from tradition is the statement that paying patients are in future to be admitted. It is within my knowledge that other Dublin hospitals have recently decided to adopt a similar policy.

### The Custom in Dublin.

This opens up a very large question. It has been the custom in Dublin as far as possible to prevent people of small means from being treated in public hospitals maintained by charitable contributions. This is as it should be. But it is also the custom for Dublin doctors to charge a minimum fee of a guinea, to cover, if necessary, three visits. A guinea is often a serious expenditure for thousands of people who hold their heads high, and, in consequence, they either refrain from taking advice in time, or else undertake to pay more than they can afford. I am stating the most elementary case. Where anything serious happens, the Dublin system produces very undesirable results. I know a man, for example, who found it necessary to undergo a minor surgical operation. His income was £500 a year, or thereabouts, and he had a family to maintain. It happened that his uncle was a doctor, whom he approached with a view to having the operation performed in a hospital supported by voluntary contributions. The uncle was a very conscientious man, and declined to help him, and delivered a lecture at the same time on the subject of charitably maintained hospitals. However, he tried another means of entry, and the operation was actually performed in the hospital at the expense of the charitable public. This seems a scandal. But I happen to know the other side. Before applying for admission, the patient made inquiry as to what it would cost to adopt the alternative method, which is to pay a surgeon, and take a bed in a nursing home. This worked out at £60, and, according to his statement, which is probably accurate, he would have had to go in debt in order to pay the fees. In a word, the city clerk or poor professional man, with an income of £250 or less, is constantly faced with the alternative of being crippled by doctor's fees or going *in forma pauperis* to a public hospital for treatment.

### A System which Demoralises.

Let it not be supposed that I am charging Dublin medical men with being sharks or bloodsuckers. They are, on the whole, broad-minded, philanthropic, and generous. But they have adopted a system which in its operation demoralises a very worthy and deserving section of the public. None of them employs an assistant: their fees are standard charges, and when people are sick they find it necessary, if they are living up to their incomes, to make "a poor mouth." To apply for admission to a public hospital is to become the object of common gossip. To enter a nursing home, very often the property of a medical syndicate, is to agree to pay, week by week, the entire income of the breadwinner. And this is merely for maintenance. In addition are the family doctor's fees, fees for consultation, surgeons and anæsthetists' fees. A pretty bill when it is all added up! It is no exaggeration to say that the horror of sickness is as nothing in comparison with the horror of the cost of treatment.

### Too Many Small Hospitals.

The whole system calls loudly for reform. In Dublin there are too many small hospitals, most of them badly equipped and miserably poor. None of them has an Almonry Department. In Belfast there are only two hospitals, but they are large and in every respect up-to-date. The refusal of the Government to extend the House of Industry grant has been instrumental in throwing this institution on its own resources. Sink or swim! The new departure indicates the dawn of intelligence. The next step will probably be to pay the medical staff, whose services are gratuitous. In the future a poor man may be able to afford to be sick. My advice to the House of Industry governors is to spread out, enlarge, and extend. You have your £7,600 from the State, and it is not to be despised. Break through the traditions and cater for the multitude. The Dublin medical profession, too, will probably discover that it is in their financial interests to abandon their time-honoured tradition of a guinea or nothing. Many of them have already arrived at this conclusion. But they are afraid to say so.

IERNE.

